# Patterns of autism symptoms: hidden structure in the ADOS and ADI-R instruments

**DOI:** 10.1038/s41398-020-00946-8

**Published:** 2020-07-30

**Authors:** Jérémy Lefort-Besnard, Kai Vogeley, Leonhard Schilbach, Gaël Varoquaux, Bertrand Thirion, Guillaume Dumas, Danilo Bzdok

**Affiliations:** 1 Department of Psychiatry, Psychotherapy, and Psychosomatics, RWTH Aachen University, Aachen, Germany; 2 Jülich Aachen Research Alliance (JARA)—Translational Brain Medicine, Aachen, Germany; 3 Max-Planck Institute of Psychiatry, Munich, Germany; 4 Department of Psychiatry and Psychotherapy, University of Cologne, Cologne, Germany; 5 Parietal Team, INRIA, CEA, University Paris-Saclay, Gif-sur-Yvette, France; 6 Human Genetics and Cognitive Functions University Paris Diderot, Sorbonne Paris Cité, Paris, France; 7 CNRS UMR3571 Genes, Synapses and Cognition, Institut Pasteur, Paris, France; 8 Human Genetics and Cognitive Functions Unit, Institut Pasteur, Paris, France; 9 Department of Biomedical Engineering, McConnell Brain Imaging Centre (BIC), Montreal Neurological Institute (MNI), Faculty of Medicine, McGill University, Montreal, Quebec Canada; 10 Mila—Quebec Artificial Intelligence Institute, Montreal, Quebec Canada

**Keywords:** Autism spectrum disorders, Psychiatric disorders

## Abstract

We simultaneously revisited the Autism Diagnostic Interview-Revised (ADI-R) and Autism Diagnostic Observation Schedule (ADOS) with a comprehensive data-analytics strategy. Here, the combination of pattern-analysis algorithms and extensive data resources (*n* = 266 patients aged 7–49 years) allowed identifying coherent clinical constellations in and across ADI-R and ADOS assessments widespread in clinical practice. Our clustering approach revealed low- and high-severity patient groups, as well as a group scoring high only in the ADI-R domains, providing quantitative contours for the widely assumed autism subtypes. Sparse regression approaches uncovered the most clinically predictive questionnaire domains. The social and communication domains of the ADI-R showed convincing performance to predict the patients’ symptom severity. Finally, we explored the relative importance of each of the ADI-R and ADOS domains conditioning on age, sex, and fluid IQ in our sample. The collective results suggest that (i) identifying autism subtypes and severity for a given individual may be most manifested in the ADI-R social and communication domains, (ii) the ADI-R might be a more appropriate tool to accurately capture symptom severity, and (iii) the ADOS domains were more relevant than the ADI-R domains to capture sex differences.

## Introduction

Autism-spectrum disorder (hereafter “autism”) is clinically defined by different types of symptoms: deficits in communication and social interaction, as well as restricted, repetitive patterns of behavior, interests, or activities. The authors of Diagnostic and Statistical Manual-5 (DSM-5)^1^ have defined autism as an umbrella term for different clinical entities in the autistic spectrum substantiated by empirical findings showing that neither clinically nor neuropsychologically different subdiagnoses could be clearly demarcated from each other^2,3^. Whereas the differentiation between clinical subgroups was skipped, the differentiation between degrees of severity has been newly included. This reintroduces the research question how specific subgroups, e.g., based on severity, may map onto symptom presentations^4,5^.

This evaluation involves a team of well-trained professionals and integrates information about the individual from different sources. Two clinical tools have become established in this assessment procedure, and are considered to be the “gold standard” in symptom evaluation for autism, particularly when combined with clinical judgment: (i) the Autism Diagnostic Interview-Revised (ADI-R)^6^, a semistructured interview conducted with parents, which focuses on current presentation and lifelong developmental history, and (ii) the Autism Diagnostic Observation Schedule (ADOS)^7^, a standardized semistructured diagnosis assessment conducted through one-to-one personal interaction and direct observation of an individual suspected to have autism using a range of activities and delivered by a trained examiner. A combination of ADI-R and ADOS assessments coupled with experienced clinical judgments has previously been shown to improve diagnostic validity^8^. In everyday clinical practice, however, often only one of the instruments is used due to time, cost, or expertise constraints. The choice of preferred instrument used for the evaluation of autism symptoms remains quite variable from one center to another.

The inconsistency in the choice of symptom-assessment tool used might have contributed to discrepancies in the results and conclusions of several studies^9,10^. Only a handful of studies have directly compared the ADI-R and ADOS, and the outcomes are not always directly clinically applicable. For instance, De Bildt et al.^11^ found that the level of agreement between the ADI-R and ADOS was slightly higher than the chance level to classify individuals as patients with autism, patients with pervasive developmental disorder, or controls. In addition, Mazefsky and Oswald^12^ found a good agreement between team diagnoses and the instruments. As such, the commonalities and divergences between the widespread ADI-R and the ADOS are yet to be fully understood.

The use of these two symptom-assessment tools requires long training, and each of them is rather time-consuming. These circumstances can lead to massive delays in diagnosis and unequal coverage of the population in need of medical attention^13^. As a consequence, delays in the delivery of therapies occur frequently, which contributes to parental distress^14,15^, and may also affect the long-term outcomes of early interventions^16^. Understanding the relatively more important factors underlying symptom severity as captured by two most often used instruments can provide some assistance to these everyday procedures on the clinical ward. Providing valuable information by capturing variability indexed by both instruments can also provide additional information for research purpose. For example, such findings offer guidance in assessing symptom severity in an efficient and accurate fashion, and could lead to significant benefits in studies where a structured scoring is required or in comorbidity investigations. Such gains would include saving patients’ time and potentially allowing for fast early intervention, reduction of clinician work hours, and alleviating economic costs, to name a few. Furthermore, given that oftentimes, only either the ADI-R or the ADOS is used for the evaluation of symptom severity in clinical practice, it is of interest to explore the impact of choosing one instrument over the other.

In view of potential benefits from refining the severity-detection process and the need of a more direct comparison of each instrument, we simultaneously revisited the ADI-R and ADOS with a comprehensive data-analytics strategy. Variability in symptoms and severity within the autism population suggests the possibility of dimensional profiles of autism symptoms. Therefore, we extracted distinct homogeneous patient-symptom profiles from the ADI-R and ADOS, giving further insights on how symptom presentation might outline different patient subgroups. In addition, we used a model that automatically selected subsets of the two instruments that are relatively most highly informative about the symptom severity. Finally, we quantitatively characterized the relation between the two instruments in the context of different patients’ age, sex, and fluid IQ (FIQ). Overall, such empirical evaluation of the ADI-R and ADOS can help disentangle effects that might have been easy to overlook otherwise.

## Methods

### Data resources

We systematically charted the relationship between the ADI-R and the ADOS based on behavioral data from a publicly available dataset: ABIDE (Autism Brain Imaging Data Exchange: https://fcon_1000.projects.nitrc.org/indi/abide/). All data analyses were carried out on this data repository, which is described in detail in Di Martino et al.^17^. The ABIDE data provide subject information, including age, sex, measures of intellectual functioning, and measures of symptom severity as assessed by the ADOS as well as the ADI-R. The behavioral assessments were collected from a total of 266 patients with an autism-spectrum disorder diagnosis, aged 7–49 years, including 233 male and 33 female subjects (see Supplementary Table 1 for details). Only the subjects that have complete information mentioned above were included in this study. The autism diagnoses were provided by board-certified psychiatrists. The distribution of the responses in ADI-R and the ADOS assessment in our sample was homogeneous (Supplementary Fig. 1). Homogeneous distribution of the ADI-R and ADOS scores was also found across patients’ age (Supplementary Fig. 2). The original studies included in ABIDE received approval from each site’s Institutional Review Board.

### Identifying hidden group structure: k-means clustering

To explore distinct subgroups among individuals with autism, we applied a k-means clustering algorithm to automatically partition patient-symptom profiles into homogeneous groups. This method is particularly useful to discover hidden factors of variation across the measured instrument domains within a subject sample or clinical population. We used “NbClust”^18^ as an established R package to simultaneously apply 30 cluster-validity metrics. This approach provided complementary ways to reach indications of the number of groups most supported by the patient data. Among all metrics of cluster usefulness, and according to the majority rule, the best number of clusters was three (see Supplementary Table 2 for the output of the NbClust package in R). That is, the most robust number of patient-symptom clusters consisted of three groups, to the extent supported by our data. Therefore, three subgroups of patients were automatically extracted in a bottom-up fashion as indicative of a more optimal underlying representation of factors hidden in the ADI-R and ADOS instruments.

### Identifying predictive relevance of domains of the ADI-R and ADOS: sparse logistic regression

The goal behind k-means was to partition the patients into nonoverlapping homogeneous groups^19^ as measured by the ADI-R and ADOS domains to explore the relationship among autism-spectrum disorder patient’s symptomatology. Complementing these insights in the next step, we applied a modeling technique that emphasizes prediction performance with an optimal trade-off against the number of the most relevant domains. That is, our model automatically picked the most relevant items among the full set of ADI-R and ADOS domains with the goal to achieve the best-possible prediction of autism severity.

To extract the most informative subsets of domains for predicting autism severity, we capitalized on the pattern-analysis algorithm sparse logistic regression^20^. Unlike the common logistic regression, the sparse logistic regression variant has an additional constraint, which, calibrated by the hyperparameter *λ*, exerts control over the parsimony criterion (i.e., optimal prediction accuracy with as few predictor variables as possible). Using nested cross-validation, the member in the model family that yielded the highest prediction accuracy (i.e., generalization performance) for each candidate of *λ* was selected. In other words, the goal here was not to select the best hyperparameter. Rather, we charted a space of candidate λ to explicitly investigate the parsimony trade-off from imposing high to low sparsity. In this way, the quantitative investigation detected subsets of domains that were most informative about the autism severity. Note that symptom modeling was carried out in an analogous fashion in previous studies^21,22^.

### Predicting age, sex, and FIQ based on the ADI-R and ADOS domains

In addition to looking for hidden relationships and the relatively most predictive variables, we wanted to extract information about which domain of the ADOS and ADI-R would be specifically informative about the patient’s age, sex, or FIQ, respectively. We therefore analyzed the relative importance of each ADI-R and ADOS domain for predicting sex, age, and FIQ using a logistic regression (without sparsity constraint). Note that we refrained from computing more sophisticated regressions with nonlinear interaction terms because these model extensions would require more than twice the sample size to obtain model fits of equal quality. Subgroups representative of each category were extracted (Supplementary Table 3). We then looked for the score of each instrument domain to predict the outcome of interest. The age and FIQ outcomes were defined as categorical summaries of the constituent continuous scores. That is, the patients’ age was defined as superior or inferior to 18 years, while the patients’ level of FIQ was defined as higher or lower than the median split of the FIQ scores.

In the first set of analyses, the sample was subdivided based on FIQ and sex. That is, four subgroups were extracted depicting males with high FIQ, males with low FIQ, females with high FIQ, and females with low FIQ. A logistic regression was applied to predict the age based on the domain scores of the ADI-R and ADOS of each specific subgroup.

For the second set of analyses, the sample was subdivided based on FIQ and age. Thus, we ended up with four subgroups, including adults with high FIQ, adults with low FIQ, teenagers with high FIQ, and teenagers with low FIQ, respectively. A logistic regression was applied to predict the sex based on the scores of the ADI-R and ADOS domains of each specific subgroup.

For the third set of analyses, the patient sample was subdivided based on sex and age. This subdivision led to four subgroups, including adult males, adult females, teenager males, and teenager females, respectively. A logistic regression was applied to predict the FIQ based on the scores of the ADI-R and ADOS domains of each specific subgroup.

For each of these analyses, in each subgroup, the logistic regression was applied only if more than ten observations were available. Furthermore, class imbalance, if present, was handled using upsampling if the majority class was inferior or equal to a third of the minority class or using downsampling otherwise. Once the weights of the logistic regression for a subgroup were computed, the bootstrapped 90% confidence intervals were calculated by fitting the logistic regression to 100 bootstrapped samples made from the specific subgroup. Finally, we computed the confusion matrix for each classification algorithm to allow visualization of its performance.

## Results

### Properties of patient groups hidden in the ADI-R and ADOS assessments

**Fig. 1:**
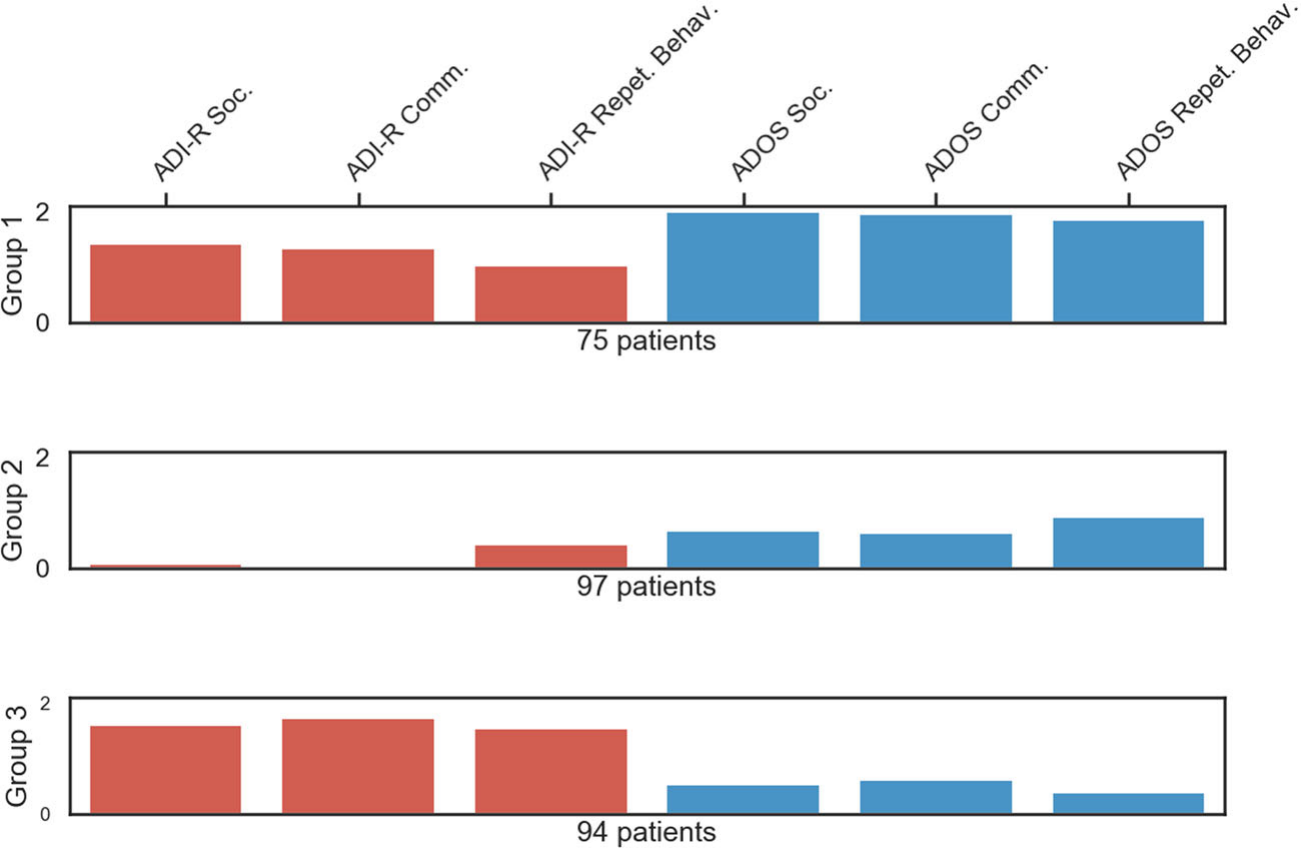
Uncovering three patient groups with distinct symptom profiles. Automatic clustering based on the domains of the Autism Diagnostic Interview-Revised (ADI-R) and Autism Diagnostic Observation Schedule (ADOS) instruments exposed three distinct symptom constellations that grouped the patients. Each row represents one data-derived symptom group constellation hidden in the patient assessments. The mean scores (*y* axis) of each domain (*x* axis) indicated the relative importance of the domains for a particular group in the k-means model. The *red* bars are the mean domain scores of the ADI-R in the respective cluster, and the blue ones are the domain scores of the ADOS. Three different autism subtypes emerged: (i) a severe profile including patients scoring high on every domain of the ADI-R and ADOS with particularly high scores on the domains of the ADOS (group 1), (ii) a mild profile including patients scoring low in every domain of the ADI-R and ADOS with particularly low scores on the social and communication domains of the ADI-R (group 2), and (iii) an ADOS-negative profile including patients scoring high in every domain of the ADI-R and scoring low in every domain of the ADOS (group 3). These findings from automatic clustering provide additional evidence that the ADI-R and ADOS questionnaires capture some distinct clinical aspects of patients with autism. Furthermore, our results suggest that the symptom severity of the ADOS-negative group might be more accurately measured by the ADI-R.

To explore distinct subgroups related to the ADI-R and ADOS-assessment patterns among patients with autism, we assigned each patient to one dominant symptom constellation based on the two instruments. This data-driven exploration revealed three distinct symptom constellations that grouped the patients in our sample (Fig. 1): (i) a severe group that included patients scoring high on every domain of the ADI-R and ADOS. Specifically, these patients had on average particularly high scores on the domains of the ADOS. (ii) A mild group that included patients who scored low in every domain of the ADI-R and ADOS. By contrast, this coherent subgroup of patients with similar profiles exhibited particularly low scores on the social and communication domains of the ADI-R. (iii) An ADOS-negative group that included patients who scored high in every domain of the ADI-R and low in every domain of the ADOS. In sum, the divergence between the social and communication domains as assessed by the ADI-R and the ADOS were the most informative markers. In contrast, the least instructive marker was the repetitive behavior domain of the two instruments (Supplementary Fig. 3). The three subgroups were homogeneous in terms of sex, age, and FIQ (Supplementary Table 4).

As an exploratory pattern-discovery approach, k-means yields clusters as a descriptive summary of our data, without primary concern for predictive validity^23,24^. A natural next step of the present study therefore consisted in estimating the predictability of autism from ADI-R and ADOS instrument domains.

### Isolating the most predictive domains in the ADI-R and ADOS instruments

**Fig. 2:**
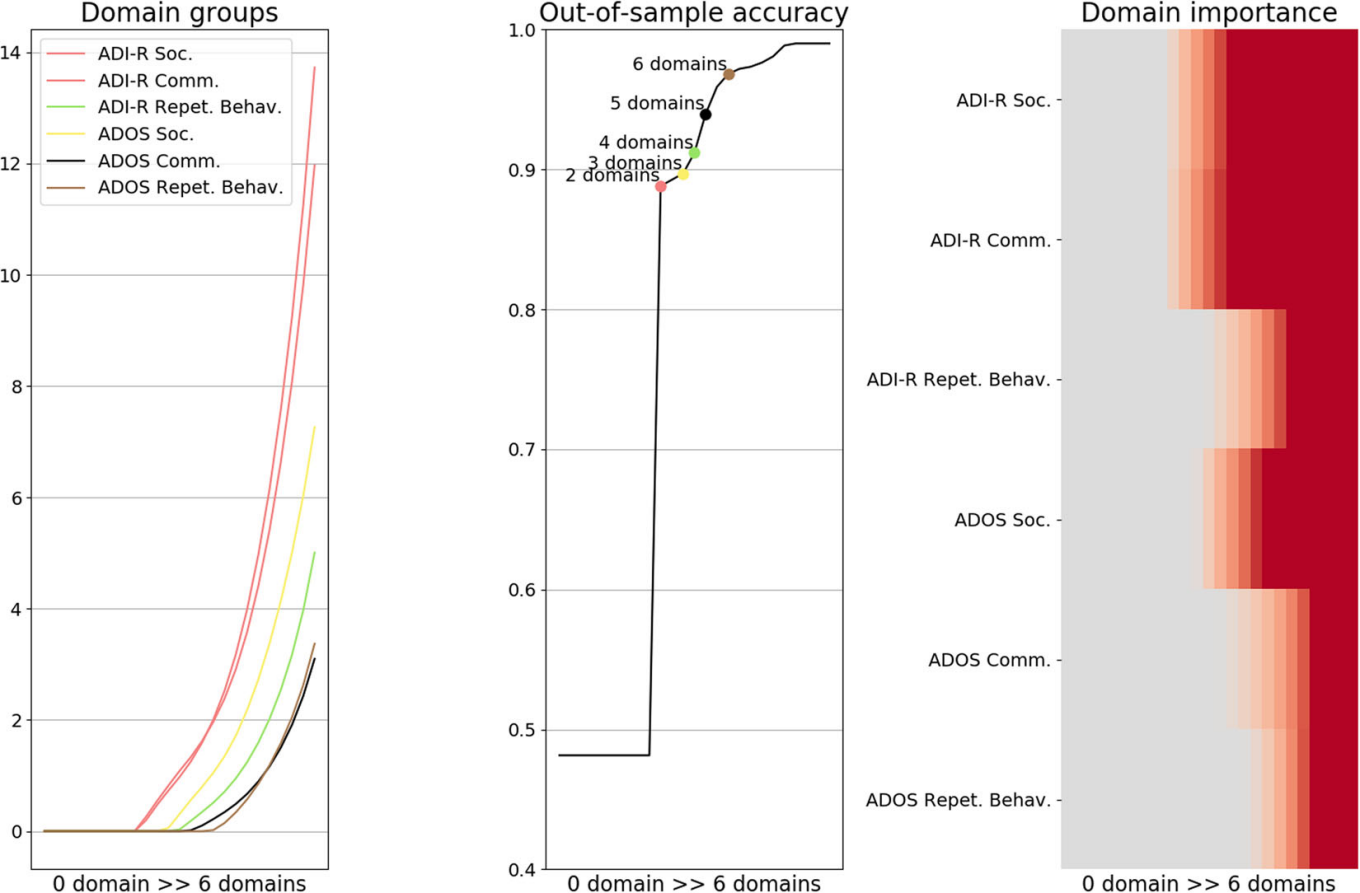
Predictive decomposition of autism symptoms. A parsimony-inducing pattern-analysis algorithm was used to search through the array of questionnaire domains and extract the most informative subsets of domains for predicting symptom severity in individuals with autism. **a***Domain groups*: Trajectories of the classifier weights of the Autism Diagnostic Interview-Revised (ADI-R) and Autism Diagnostic Observation Schedule (ADOS) domains are plotted on the *y* axis, while the parsimony constraint of the statistical models decreasing from left to right (here represented as the increasing number of domains automatically selected) is plotted on the *x* axis. The curves indicate changes in the subset of selected domains (i.e., the weight set not equal to zero), typically an inclusion. The color of each line shows in which model solution a specific questionnaire domain is included as relevant. For example, ADI-R social and communication domains were found as part of the first (most parsimonious) predictive model and are plotted in red. **b***Prediction accuracy*: The middle panel retraces how prediction performance increases step by step as the identified domain subsets are added to the model. Each colored point represents a predictive model, including a specific number of selected domains. Two domains were sufficient for decent prediction performance at the single-subject level. These two domains predicted autism severity with 88.81% accuracy, while the model including every domain of the ADI-R and ADOS predicted autism severity with 96.81% accuracy. **c***Relative domain importance*: Domain importance in the active weights is indicated as the parsimony constraint that becomes more lenient (left to right). This panel thus represents the relative importance of each domain (*y* axis) as more variables are included in the model (*x* axis, from left to right). In sum, the results emphasize that using every domain of the ADI-R and ADOS, autism severity was predicted with 97% accuracy; while using only the social and communication domains of the ADI-R, autism severity was predicted with an accuracy as high as 89%, indicating a very high predictive power for these two elements.

A sparse logistic regression was used to automatically identify domain subsets in the ADI-R and ADOS instruments that are most informative about telling mild versus severe autism apart in potentially new patients. With systematically varying parsimony constraint, a series of algorithm estimations was carried out to predict autism severity (defined as the median split of the ADI-R and ADOS total score) based on the symptom scales (Fig. 2a, c).

Our analysis strategy extracted two of the overall six domains as the most predictive subset and achieved quite effective prediction of autism severity (88.81% accuracy). This essential subset included the social and communication domains of the ADI-R. Note that we obtained virtually identical results in a supplementary analysis exploring more sophisticated domain–domain relationships using random-forest algorithm (see Supplementary Methods, Results, and Discussion and Supplementary Fig. 4).

As we calibrated the parsimony constraint step by step, four other solutions were automatically identified that isolated further subsets of instrument domains predictive of autism severity (Fig. 2b). The second solution included the social domain of the ADOS in addition to the social and communication domains of the ADI-R, and reached a prediction accuracy of 89.7%. The three remaining automatically identified solutions successively incorporated into the previous subset the repetitive behavior domain of the ADI-R, the communication domain of the ADOS, and finally included the repetitive behavior of the ADOS. These three final domain inclusions allowed for a prediction accuracy of 91.18%, 93.96%, and 96.81% in new patients, respectively. In other words, rich descriptions of the patterns in the ADI-R and the ADOS were extracted, and two domains of the ADI-R were identified as being highly predictive of autism severity. In sum, though the combination of both ADI-R and ADOS instruments is required for a more rigorous severity-estimation procedure, our data-guided analysis gave support to the existence of a subset, including two ADI-R domains highly predictive of autism severity.

### Exploring the instrument domains’ relations to patients’ age, sex, and FIQ

**Fig. 3:**
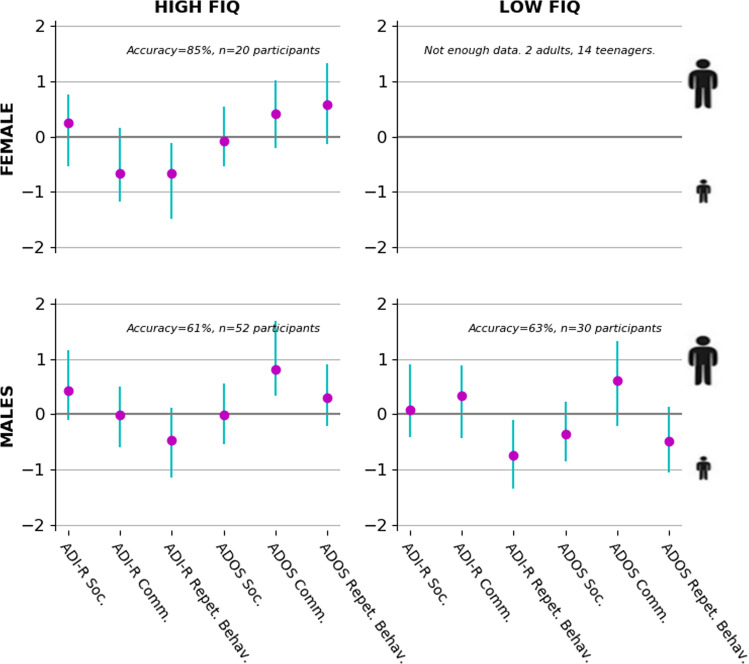
Domain importance in age prediction segregated by sex and fluid IQ (FIQ). Adult versus teenager was distinguished based on domains quantified in the Autism Diagnostic Interview-Revised (ADI-R) and Autism Diagnostic Observation Schedule (ADOS). Each plot shows identical analyses on a stratified subset of the patient pool. From upper left to lower right, we selected the male patients with high or low FIQ, and the female patients with high FIQ. We had insufficient female patients with low FIQ to perform the analysis. The purple circles show the estimated contribution (*y* axis) of each particular questionnaire domain (*x* axis) to distinguishing adult versus teenager participants using logistic regression in each specific subgroup. Each green bar indicates the bootstrapped 90% uncertainty interval at the population level. The communication domain of the ADOS contributed to detecting an adult in our sample, while a patient scoring high in the repetitive behavior of the ADI-R was more likely to be a teenager. In other words, a patient scoring high in the communication domain of the ADOS would tip the balance of the output toward being an adult, while scoring high in the repetitive behavior domain of the ADI-R would tip it toward being a teenager. In females with high FIQ, the communication domain of the ADI-R was most associated with being a teenager. In males with high FIQ, the social domain of the ADOS contributed to detecting a teenager, while in males with low FIQ, the communication domain of the ADI-R contributed to detecting an adult. Finally, the repetitive behavior domain of the ADOS was contributing to detecting male and female adults with high FIQ, while this ADOS domain was contributing to detecting a teenager in males with low FIQ.

In the first set of analyses, to quantify the relation of the ADI-R and the ADOS domains with their relation to age, we explored the contribution of each instrument domain in deriving age of patients in our sample. Age (i.e., target variable) was predicted based on the ADI-R and ADOS domains (i.e., input variables) after segregating the patient pool into subgroups according to sex and FIQ (Fig. 3; Supplementary Fig. 5). For this analysis and the following ones, only the domains with important weight per estimation and exhibiting mostly one-sided confidence intervals were interpreted (Supplementary Table 5). In this first analysis, the communication domain of the ADOS contributed to detecting an adult, while the ADI-R repetitive behavior domain contributed to detecting a teenager in each subgroup. In other words, a patient scoring high in the ADOS communication domain would tip the balance of the output toward being an adult, while scoring high in the ADI-R repetition behavior domain would tip it toward being a teenager. In females with high FIQ, the ADI-R communication domain was highly weighted contributing to being a teenager. In males with high FIQ, the social domain of the ADI-R contributed to detecting an adult, while in males with low FIQ, the social domain of the ADOS contributed to detecting a teenager. Finally, the repetitive behavior domain of the ADOS was contributing to being an adult in males and females with high FIQ, while this ADOS domain was contributing to being a teenager in males with low FIQ.

**Fig. 4:**
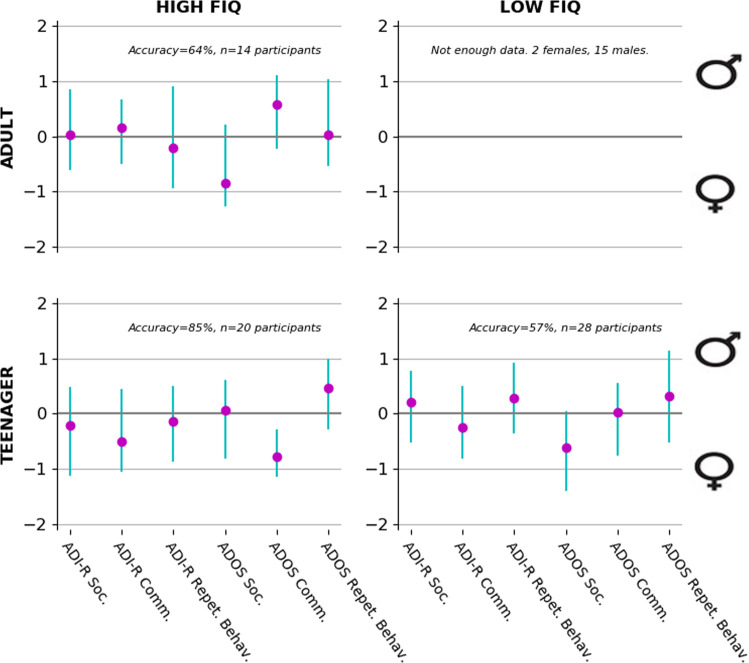
Domain importance in sex prediction segregated by age and fluid IQ (FIQ). Male versus female patients were distinguished based on domains quantified in the Autism Diagnostic Interview-Revised (ADI-R) and Autism Diagnostic Observation Schedule (ADOS). Each plot shows identical analyses on a stratified subset of patient pool. From upper left to lower right, we selected the adult patients with high FIQ, and the teenager patients with high or low FIQ. We had insufficient adult patients with low FIQ to compute the analysis. The purple circles show the estimated contribution (*y* axis) of each particular questionnaire domain (*x* axis) to distinguishing male versus female participants using logistic regression in each specific subgroup. Each green bar indicates the bootstrapped 90% uncertainty interval at the population level. The ADI-R domains hardly contributed to differentiating sex. The social domain of the ADOS was highly associated with detecting a female in both adults with high FIQ and teenagers with low FIQ. The communication domain of the ADOS enabled detecting a male in adults with high FIQ and a female in teenagers with high FIQ. The repetitive behavior domain of the ADOS was highly weighted in teenagers with high FIQ, contributing to detecting males.

In the second set of analyses, to quantify the relation of the ADI-R and the ADOS domains with their relation to sex, we explored the contribution of each instrument domain in deriving the sex of patients in our sample. Sex (i.e., target variable) was predicted based on the ADI-R and ADOS domains (i.e., input variables) after segregating the patient pool into subgroups according to age and FIQ (Fig. 4; Supplementary Fig. 6). The ADI-R was not contributing for differentiating sex. The ADOS social domain was highly weighted, contributing to identifying females among both adults with high FIQ and teenagers with low FIQ. The ADOS communication contributed to detecting a male among adults with high FIQ, and to being a female among teenagers with high FIQ. The ADOS repetitive behavior domain was highly weighted among teenagers with high FIQ contributing to being a male.

**Fig. 5:**
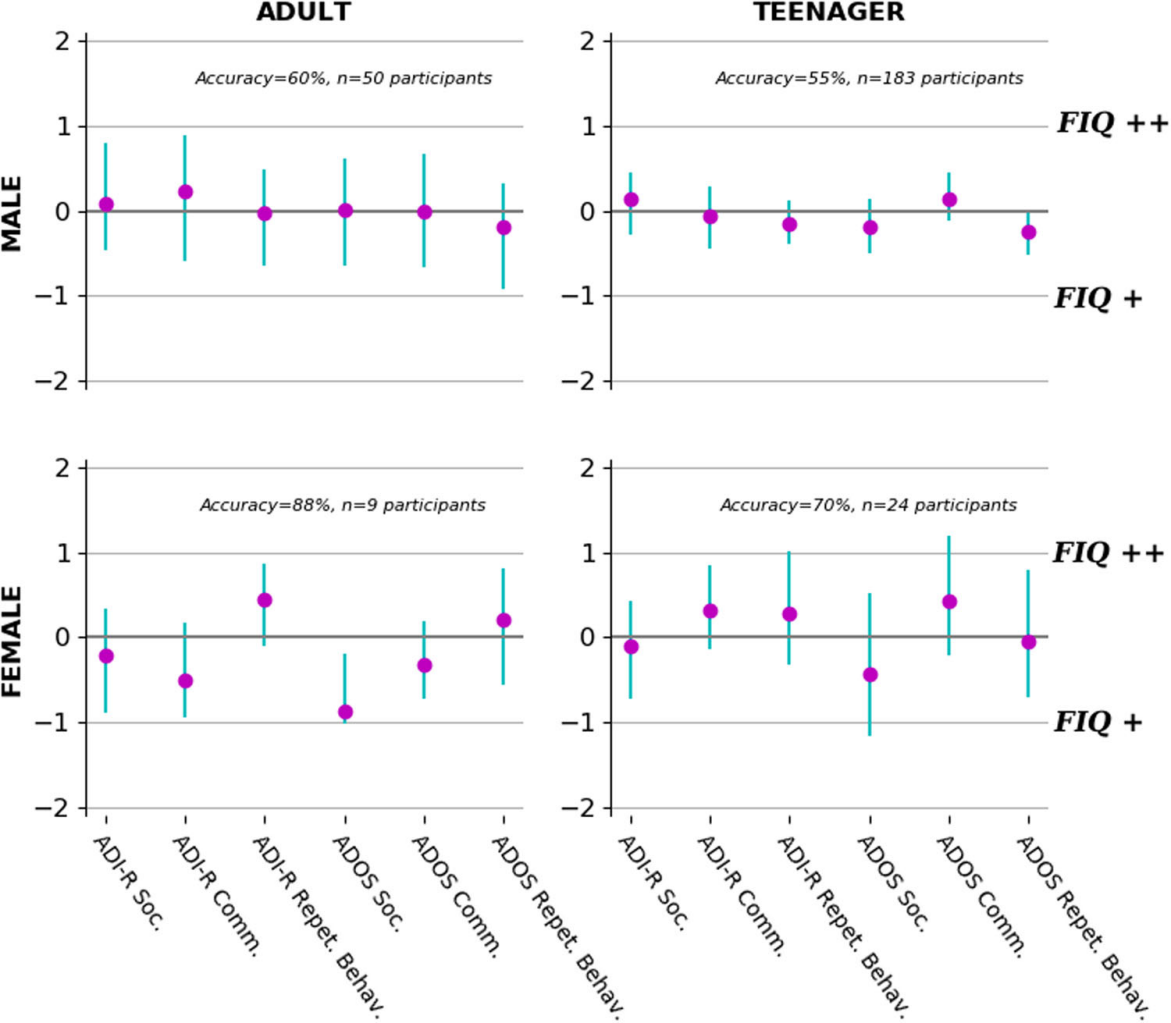
Domain importance in fluid IQ (FIQ) prediction segregated by sex and age. High versus low FIQ was distinguished based on domains quantified in the Autism Diagnostic Interview-Revised (ADI-R) and Autism Diagnostic Observation Schedule (ADOS). Each plot shows identical analyses on a stratified subset of the patient pool. From upper left to lower right, we selected the adult or teenager male patients, and the adult or teenager female patients. The purple circles show the estimated contribution (*y* axis) of each particular questionnaire domain (*x* axis) to distinguishing participants with high versus low FIQ using logistic regression in each specific subgroup. Each green bar indicates the bootstrapped 90% confidence interval at the population level. In teenager males, the repetitive behavior of the ADI-R, the social, and repetitive behavior domains of the ADOS were contributing to having a low FIQ, while the communication domain of the ADOS helped detecting high FIQ. In adult females, the communication domain of the ADI-R and the social domain of the ADOS contributed to detecting a low FIQ, while the repetitive behavior domain of the ADI-R contributed to detecting a high FIQ. In teenager females, the communication and repetitive behavior domains of the ADI-R, as well as the communication domain of the ADOS, were highly associated with detecting high FIQ. Interestingly, the domains of the ADI-R and ADOS were not informative of the patients’ level of FIQ in adult males.

In a final set of analyses, to quantify the relation of the ADI-R and the ADOS domains with their relation to FIQ, we explored the contribution of each instrument domain in deriving the level of FIQ of patients in our sample. FIQ (i.e., target variable) was predicted based on the ADI-R and ADOS domains (i.e., input variables) after segregating the patient pool into subgroups according to age and sex (Fig. 5; Supplementary Fig. 7). In teenager males, the repetitive behavior domain of the ADI-R as well as the social and repetitive behavior domains of the ADOS were contributing to having a low FIQ, while the ADOS communication domain contributed to detecting a high FIQ. In adult females, the communication domain of the ADI-R as well as the social domain of the ADOS contributed to detecting a low FIQ, while the repetitive behavior domain of the ADI-R contributed to detecting a high FIQ. In teenager females, the communication and repetitive behavior domains of the ADI-R as well as the communication domain of the ADOS were highly weighted, contributing to having a high FIQ.

Across analyses, our results show that the communication domain of the ADOS was the most salient overall domain in distinguishing sex, age, and FIQ (seven contributions), and the ADOS domains were the most informative about the patient’s sex.

## Discussion

Our study emphasizes the relatively bigger importance of certain parts of two widely administered clinical instruments for autism. The social and communication domains of the ADI-R were found particularly informative for predicting symptom severity confirming the informative value of these domains in our sample. Further, three dimensional partitions of discrete autism profiles were uncovered, and these three subgroups exhibited the highest discrepancy on the social and communication domains of the two instruments. In addition, we provide a comprehensive characterization of how much information each instrument domain carries about the patients’ age, sex, and FIQ. Collectively, our results provide quantitative insights into the relation between ADI-R and ADOS by using a set of modern data-analysis tools.

### Extracting the most predictive domain subsets in the ADI-R and ADOS

As a primary focus of the present investigation, we algorithmically identified the most predictive ADI-R and ADOS domains of symptom severity. Given that each examined domain contains information about some aspect of symptom severity, we explored a more subtle question: To what extent is each domain relative and more or less informative, compared to each other, and which domain subset would be sufficient for assessing individuals’ symptom severity? Using all domains from the ADI-R and ADOS, the sparse logistic regression predicted symptom severity with an accuracy of 97%. In particular, the first solution that gave nonzero coefficients was a subselection of 2 domains that predicted symptom severity with an accuracy of about 89%, thus only 8% below the accuracy obtained using all domains. The specific subset included the social and communication domains of the ADI-R. This core subset of instrument domains was highly predictive of symptom severity.

More generally, the ADI-R and ADOS assessments are often mentioned to be well designed and comprehensive, but not to be particularly efficient to administer in clinical practice. In fact, ~45 min are required for the ADOS assessment, while ~1.5 h are required for the ADI-R assessment. Furthermore, extensive training is needed to use each instrument, which makes the combined assessment very heavy and time-expensive. Berument et al.^25^ found that the diagnostic accuracy as well as the estimation of symptom severity based on the ADI was pretty much the same as a 40-item scale, suggesting that a much shorter and highly effective screening instrument can be created. In a similar spirit, our results show that assessing only two domains from the ADI-R assessment may be sufficient for making reliable statements about the rough overall severity of the psychopathology of patients with autism. In sum, although the combination of ADI-R and ADOS assessments has been shown to improve autism diagnostic validity, we found support for a subset highly predictive of symptom severity in a data-guided fashion nevertheless.

Mazefski et al.^26^ highlighted a discrepancy in the ability of the ADOS to capture autism symptoms cataloged in the DSM-5. These authors found that the ADI-R was more relevant than the ADOS for tapping on the breadth of autism symptoms as defined by the DSM-5. Similarly, Wiggins and Robins^27^ reported that using only the social and communication domains of the ADI-R resulted in improved sensitivity and specificity of the instrument. Our results lend support to these previous findings in showing that the social and communication domains of the ADI-R were more predictive of symptom severity than any other domain from the ADOS, and therefore potentially relevant for an effective screening of symptom severity.

In sum, our results lend support to the predictive value of the derived instrument subset, comprising the social and communication domains of the ADI-R. From a clinical perspective, being able to find evidence for repetitive behavior has often been considered as being indicative of an autism diagnosis, while only finding interaction and communication difficulties is considered less convincing^28^. This stands in contrast to our empirical findings that emphasize the predictive value of the interaction and communication domains. This observation adds further arguments in favor of assisting medical decision-making in psychiatry by a predictive machine-learning algorithm toward the future of precision medicine. Finally, it is important to note that the ADI-R and ADOS measures of symptom severity might not always be necessary or informative. For example, patients presenting severe symptoms in early life may not in all cases need to receive a scored diagnosis. Such clinical instruments see widespread use, especially for research purposes. However, in such setting, a summary score based on the clinical instruments as severity reflection can be of value. Our results helped highlight key components of these instruments, and thus encourage the reconciliation and integration of the ADI-R and ADOS by providing quantitative insights into the relation between the two instruments. Our results uncovered principles that can help guide the choice of a most suitable instrument when time constraints prompt the medical team to use only one assessment tool, rather than several ones.

### Extracting patient subgroups from the ADI-R and ADOS

Given that autism is widely acknowledged to be a spectrum disorder, we aimed at providing windows into useful intermediate phenotypes. Using a clustering algorithm, three distinct types of clinically meaningful symptom sets emerged, each underlying set being summarized with its average domain scores: (i) a severe profile with high expression in each domain of the ADI-R and ADOS, (ii) a mild profile with low scores on both instruments with particularly low associations with the social and communication domains of the ADI-R, and (iii) an ADOS-negative profile with high scores only in the domains of the ADI-R. In each subgroup, the social and communication domains of both instruments were the most informative markers, while the repetitive behavior domain of both instruments was not as informative. Our results provide data-driven evidence that a major difference between autism patients is the extent of the social and communication domain symptoms as provided by the ADI-R and the ADOS. Our analysis also revealed a group of patients scoring high only in the ADI-R. The ADOS may be used where information of childhood development is not present or judged unreliable to substantiate clinical judgment. For certain patients, symptoms might remain hidden when only the ADOS is used for the symptom-severity assessment. These patients are sometimes classified as ADOS-negative^29^. This ADOS-negative subgroup suggests that the ADI-R might be a more appropriate tool to accurately capture symptom severity when assessing symptoms using both instruments is not achievable.

A few existing studies also applied a clustering method to extract meaningful information, but only from either the ADOS or the ADI-R. For instance, using k-means clustering, Cholemkery et al.^30^ found three clusters in a sample of patients assessed with the ADI-R. Their results indicated a different pattern of social interaction and communication problems versus stereotype behaviors across the identified subgroups. The characteristics of the extracted clusters fit with the assumption of a severity gradient across the three subgroups they found. Similarly, a severity gradient was the main difference between the subgroups that emerged in our sample. Spiker et al.^31^ also used k-means clustering in a sample of siblings with autism, and found subgroups that could be characterized along a single and continuous severity dimension. Children with the lowest nonverbal IQ scores exhibited higher scores in each domain of the ADI-R. Those with higher nonverbal IQs had ADI-R scores indicative of less severe impairment. Interestingly, these authors found that repetitive behavior scores were negatively correlated with this severity gradient in their sample, which corroborates our results in two ways. First, we also found a severity gradient across the three clusters mostly related to the social and communication domains of the two instruments. Given the ADOS-negative subgroup, the ADI-R social and communication domains might therefore be a good trade-off to capture symptom severity if the time constraint discourages the use of the two instruments. Second, in the three subgroups that emerged from our sample, the repetitive behavior domain of the ADI-R and ADOS expressed in the three scenarios an opposite pattern of the severity gradient. In the subgroup exhibiting the highest scores in each domain of the ADI-R and ADOS, the repetitive behavior domain had the lowest weight compared with the remaining domains of the same instrument. In the subgroup exhibiting the lowest scores in each domain, the repetitive behavior domain of the ADI-R and ADOS had the highest one compared with the two others of the same scale. Finally, in light of the release of DSM-5, which, unlike its predecessor, includes a severity ranking for the autism diagnosis, our results support the adequacy of the dimensional—as opposed to a categorical—conceptualization of the spectrum. Indeed, one of the key changes in DSM-5 is the removal of the DSM-IV autism clinical subtypes in favor of a dimensional approach. In our study, each emerged subgroup harbored a different severity gradient rather than a categorical distinction, suggesting that a dimensional approach to study the heterogeneous autism phenotype should indeed be encouraged^5,32,33^.

In sum, our results corroborate previous findings on potential autism subtypes. Furthermore, our results have repeatedly emphasized the relevance of the social and communication domains of the ADI-R, which were found to be highly informative of autism severity in our previous analyses.

### Relation of instrument domains to patients’ age, sex, and, FIQ

We explored the contribution of each instrument domain in deriving age, sex, and level of FIQ of patients in our sample. Our results suggest that (i) the ADI-R and ADOS are differently useful to uncover relevant information of patients with autism, and that (ii) sex differences might be more contrasting through the ADOS rather than through the ADI-R domains.

Some studies identified differences between girls and boys in social symptoms on the ADI-R^34,35^. However, no sex effects were found when the authors controlled for IQ differences^36^. Our results complemented these previous findings by suggesting that the ADI-R was not informative about the sex. A few authors have reported greater socio-communication difficulties as captured by the ADOS in females^35,37,38^. Corroborating these results, we found the social and communication domains of the ADOS to be often informative about the patient’s sex. Scoring high in the social domain of the ADOS was indicative of a female in teenagers and adults, which contrasts with the “adolescent emergence hypothesis”. This theory states that the social deficits in females appear later in time^39,40^. Sex differences should hence gradually disappear with age, which was not the case in our sample. Given that in subsets of adult patients, social and communication domains were as informative about sex differences as in subsets of teenagers, our results are also in contrast with the “female compensation hypothesis”. According to this theory, females better learn over time to compensate for their social difficulties (i.e., social camouflage) more effectively than males^40–43^, which would lead to a higher sex difference later in time. Interestingly, scoring high in the communication and social domains of the ADOS was indicative of a female in subsets of patients that were teenagers. Thus, our results suggest that social and communication symptom deficits are less visible in teenager male patients, which, again, is in contrast with the two previous hypotheses.

Our results shed light on many sex differences in the ADOS scoring. Surprisingly, the ADI-R was not very useful to differentiate patients’ sex in our sample. However, the two instruments were informative about patients’ level of FIQ and age. Indeed, interesting patterns emerged regarding the level of FIQ and age of patients. For example, we found the repetitive behavior domain of the ADI-R to be very useful to distinguish the age and the FIQ level of the patients. A patient scoring high on the repetitive behavior domain of the ADI-R was more likely to be identified as a teenager rather than an adult in every subset of patients. This pattern potentially indicates that these symptoms attenuate with age, which was also found in previous studies^44–46^. Conversely, we found that patients who scored high in the communication domain of the ADOS were more likely to be adults rather than teenagers. These results suggest that communication problems are harder to disguise as patients grow up. In contrast, previous findings from Seltzer et al.^46^ found no difference between teenagers and adults in the communication symptom severity.

In sum, our last set of analyses corroborates previous findings showing that both the ADI-R and the ADOS are differently informative about the patients’ age and level of FIQ. Our results also emphasize the higher relevance of the ADOS than the ADI-R to estimate patients’ sex.

## Conclusion

Improving the process of estimating and monitoring autism symptom severity is an emerging agenda to improve early intervention, clinical effectiveness, and reduced cost. On the one hand, our quantitative investigations expose a subset of the ADI-R domains to be sufficient for making reliable statements about the overall severity of autism symptoms, and uncovered three types of distinct, clinically meaningful patient categories. On the other hand, the discovered subgroup effects showed that patterns of ADI-R and ADOS scores discriminate patients’ sex, age, and level of FIQ. A combination of ADI-R and ADOS assessments improves the severity estimation, but worsens the already-heavy process to detect autism symptom severity. In today’s medical practice, clinical centers often use only one instrument, but the choice of preferred instrument remains quite variable. Our data-driven research identified some principles that can help guide the choice of a most suitable instrument when the time constraint prompts the medical team to use only one. Furthermore, our study paves the way to reconciling and integrating the ADI-R and ADOS. Our data repository provides information from a large sample, but may be limited to certain less well-covered demographic characteristics. Thus, replication in separate and larger datasets in future research will be important to back up our results. Nevertheless, analysis approaches based on subgroups of patients with similar characteristics may be critical to disentangle the “female compensation” and “adolescent emergence” hypotheses that have been proposed to underlie autism^34,39–42^. Given the heterogeneity among patients, studying subsets of patients with similar characteristics can provide advantages in future intervention and research in autism. Increasingly available “big data” resources will allow for such fine-grained explorations. Future studies may also include the effect of the clinician’s training and prior experience with the ADI-R and ADOS instruments as biases can occur in the interview process. Finally, note that we investigated two gold-standard instruments that are widely used in clinical practice, but we did not map the derived groups to their neurobiological manifestations. Careful description of these neural differences underlying autism spectrum disorder will provide important insights into how to make progress toward precision medicine in psychiatry.

## Supplementary information

Supplementary Information

## Data Availability

All analysis scripts of the present study are readily accessible to the reader online (https://github.com/JLefortBesnard/ADIR_ADOS2019). See Supplementary Methods for more details.
