# Supplementary material for: Patterns of autism symptoms: hidden structure in the ADOS and ADI-R instruments
Source: Transl Psychiatry. 2020 Jul 30;10:257. doi: 10.1038/s41398-020-00946-8 (PMC7393151; doi:10.1038/s41398-020-00946-8)
Supplement: Supplementary file 1 — Supplementary Information [file 41398_2020_946_MOESM1_ESM.docx]

**SUPPORTING INFORMATION APPENDIX**

**"Patterns of Autism Symptoms:**

**Hidden Structure in the ADOS and ADI-R instruments"**

Jérémy Lefort-Besnard, Leonhard Schilbard, Kai Vogeley, Gaël Varoquaux,

Bertrand Thirion, Guillaume Dumas, Danilo Bzdok

**Contents:**

**1. Supplementary methods (2 pages)**

**2. Supplementary results (1 page)**

**3. Supplementary discussion (1 page)**

**4. Supplementary tables (5 pages)**

**5. Supplementary figures (9 pages)**

**6. Supplementary bibliography (1 page)**

**1. SUPPLEMENTARY METHODS**

*Identifying predictive relevance of domains of the ADI-R and ADOS: Sparse logistic regression*

The goal behind k-means was to partition the patients into non-overlapping homogeneous groups (Bzdok & Meyer-Lindenberg, 2017) as measured by the ADI-R and ADOS domains. This approach allowed us to explore the relationship among autism spectrum disorder patient’s symptomatology. Complementing these insights in a next step, we applied a modeling technique that emphasizes prediction performance with an optimal tradeoff against the number of most relevant domains. That is, our model automatically selected the most relevant subsets of ADI-R and ADOS domains to predict autism severity.

To extract the most informative subsets of domains for predicting autism severity, we capitalized on the pattern analysis algorithm *sparse logistic regression* (Hastie, Tibshirani, & Wainwright, 2015). The sparsity constraint was imposed in form of an $l_{\text{1}}$ regularization penalty added to the objective of the generalized linear modeling problem. Such a constraint in the optimization objective automatically detects relevant features “on-the-fly” during model estimation. The $l_{\text{1}}$ penalty term, calibrated by the hyper-parameter λ, exerts control over the parsimony criterion and its shrinkage regularization on the learned model weights. The penalized negative log-likelihood of the sparse logistic regression objective is given by:

$\frac{-1}{N}\sum_{i=1}^{N} log\left( 1+e^{-y_{i}f\left( x_{i};\beta_{0},\beta\right)} \right)+\lambda\left\| \beta\right\|_{1},$

where $x_{i}$ represents a given patient’s ADI-R and ADOS domain scores, $y_{i}$ is his or her autism severity group defined as the median-split of the ADI-R and ADOS total score (i.e., 0 as mild, 1 as severe) representing a categorical summary of the constituent continuous scores, $\beta_{0}$ is the intercept, and $\beta$ is the weight attached to each instrument domain, and the right term corresponds to the $l_{1}$ penalty term controlled by the hyper-parameter λ. The domain selection behavior is calibrated by the choice of this tuning parameter. The value of λ was varied logarithmically from 3.5 to 1.0 in log-space with 16 steps. Using nested cross validation (i.e., data-dependent model validation inside of another such empirical model check)*,* the member in the model family that yielded highest prediction accuracy (i.e., generalization performance) for each candidate of λ was selected. In other words, the goal here was not to select the best hyper-parameter. Rather, we charted a space of candidate λ to explicitly investigate the parsimony tradeoff from imposing high to low sparsity. In this way, the quantitative investigation detected subsets of domains that were most informative about the autism severity. It is important to note that the pattern-learning process (i.e., the automatic procedure performing subsets selection) was independent of any knowledge about the data. That is, the algorithm automatically detected the most predictive subsets based on intrinsic properties of the questionnaire domains.

*Assessing domain importance in predicting autism severity*

The k-means method (cf. main manuscript) extracted latent structure dormant in the data regardless of symptom severity measures. Sparse logistic regression (cf. main manuscript) in turn selected the most predictive variables. Here, we wanted to make sure that the sparse logistic regression was more appropriate to our research setting than a model looking for higher-order effects. That is, a model sets out to find any interplay between each domain by intricate variable-variable interactions going beyond weighted sums of items to predict the severity. We thus compared the prediction performance of the sparse logistic regression to the accuracy reached by a commonly used non-linear predictive model: the random-forest algorithm.

The random-forest algorithm is an ensemble learning method that operates by constructing a multitude of decision trees and outputs its prediction estimate for each tree to obtain a committee decision across all trees (Breiman, 2001). However, in building a random-forest, at each split in the tree, the algorithm can only consider a random subset of all input variables (i.e., the ADI-R and ADOS domains). The square root of the total number of input variables is typically chosen (James, Witten, Hastie, & Tibshirani, 2013). Therefore, in our study, two input variables were randomly picked at each split. That is, random-forest algorithm forces each split to be based on only a random subset of two input variables. This process can be thought as decorrelating the trees and leads to reduced variance. At each decision node of the tree, the algorithm selects the two input variables that maximizes the separation between groups (i.e., mild vs severe autism). This process is continued recursively for all nodes until each leaf of the tree defines unique class. The final classification is obtained by a majority vote among all built decision trees in the ensemble. The severity of autism symptoms in a given patient was thus derived based on the most consistently predicted outcome of the built decision trees.

The random-forest algorithm derives an importance metric for each input feature called variable importance. Ultimately, variable importance demonstrates which variables have a major role in discriminating patients with a severe or mild autism. Instability of variable importance from the random-forest algorithm can happen due to sampling effects. Therefore, we computed the 90% bootstrapped confidence intervals of the variable importance by fitting a random-forest algorithm to 100 bootstrap samples made from our original sample. Bootstrapped samples can be considered as generated datasets and represent quite good approximations for population parameter (Efron & Tibshirani, 1994). In our study, bootstrapping was used to provide a principled estimate of the statistical quality of the variable importance for estimating autism severity.

*Code availability*

Python was selected as scientific computing engine. Capitalizing on its open-source ecosystem helps enhance replicability, reusability, and provenance tracking. Scikit-learn (Pedregosa et al., 2011) provided efficient, unit-tested implementations of state-of-the-art statistical learning algorithms ([http://scikit-learn.org](http://scikit-learn.org/)). All analysis scripts of the present study are readily accessible to the reader online (https://github.com/JLefortBesnard/ADIR_ADOS2019).

**2. SUPPLEMENTARY RESULTS**

*Testing for non-linearity in the ADI-R and ADOS instruments*

To complement the sparse logistic regression insights, we combined exploration of more sophisticated domain-domain relationships with the evaluation of prediction performance using random-forest algorithm. The goal here was to assess that a complex model would not reach a higher accuracy than the sparse logistic regression in predicting autism severity. Therefore, we used the random-forest algorithm to predict autism severity using the score of the ADI-R and ADOS domains. This predictive algorithm allows to evaluate the importance of features for discriminating between mild and severe clinical presentation. The 6 domains of the ADI-R and ADOS were used as input variables.

The model reached an out of sample accuracy as high as 92.78%. The social and communication domains of the ADI-R were the most informative features to distinguish the patients with autism with 38.18% and 34.17% of explained variance respectively (SFig. 4). In sum, both the random-forest algorithm and the sparse logistic regression found the social and communication domains of the ADI-R to exhibit patterns of interest in the estimation of autism severity. Random-forest reached a maximum prediction accuracy of 92.78%, while the accuracy obtained with the sparse logistic regression using every domain of the ADI-R and ADOS reached 96.81%. In other words, the social and communication domains of the ADI-R were found particularly informative for predicting autism severity when looking for additive effects as well as non-linear interactions, confirming the informative value of these domains in our sample.

**3. SUPPLEMENTARY DISCUSSION**

*Testing for non-linear relationships between the instrument domains*

A large majority of previous studies on autism have exclusively relied on applying a linear statistical model that extracts additive effects between the studied input variables. In a second analysis, we investigated the idea that more elaborate statistical relationships among instrument domains may explain the response variability among patients with autism. Therefore, we looked for interaction effects between the domains. We used random-forest algorithms to classify patients into severe versus mild groups based on their scores on the ADI-R and ADOS domains. The obtained prediction accuracy was as high as 92.78%. Chen and colleagues (2015) used intrinsic functional connectivities for predicting diagnostics of autism patients. While accuracy remained overall modest for the application of the linear approaches, they obtained a high accuracy (91%) with the application of random-forest algorithms. A more recent successful application of random-forest algorithms was carried out by Feczko and colleagues (2018). Using behavioral data from seven tasks assessing information processing, these authors classified autism with an accuracy of 73%. Similarly, our results showed that the random-forest algorithm, capturing higher-order interactions between the ADI-R and ADOS domains, predicted autism severity with a quite high accuracy.

Furthermore, the random-forest algorithm allows to estimate the importance of each feature. We therefore assessed the importance of each domain of the ADI-R and ADOS for discriminating autism severity in patients. Similar to our findings from sparse logistic regression, the social and communication domains of the ADI-R were found to be the most informative features confirming the special importance of these two domains for assessing autism severity.

In sum, using the sparse logistic regression led to a higher predictive accuracy. Additionally, our analyses suggest both additive and interaction effects for the ADI-R and ADOS domains. Furthermore, our supplementary quantitative analyses confirm the relatively higher importance of the social and communication domains of the ADI-R for the clinical assessment of autism severity.

**4. SUPPLEMENTARY TABLES**

**Supplementary Table 1:** characteristics of the sample


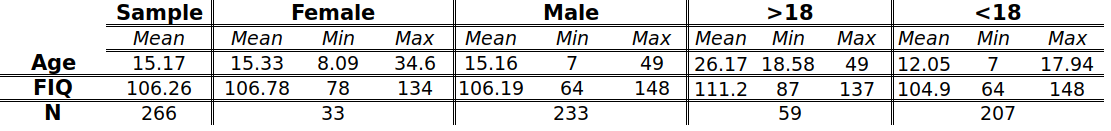


**Supplementary table 2:** output of the NbClust function in R applied in our sample


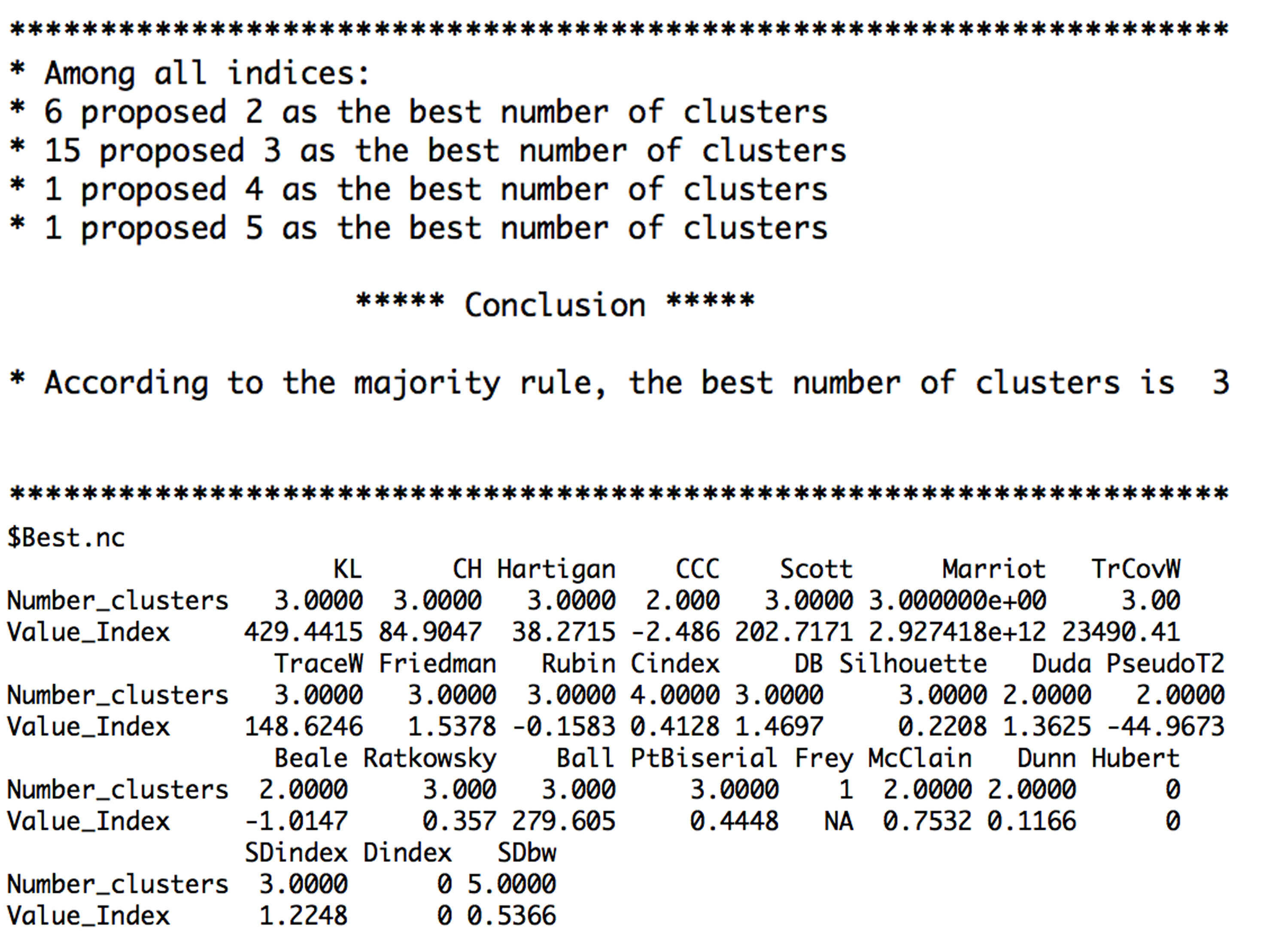


**Supplementary table 3:** Characteristics (count, mean and standard deviation) of each subgroup for the age, sex and FIQ subdivision analysis


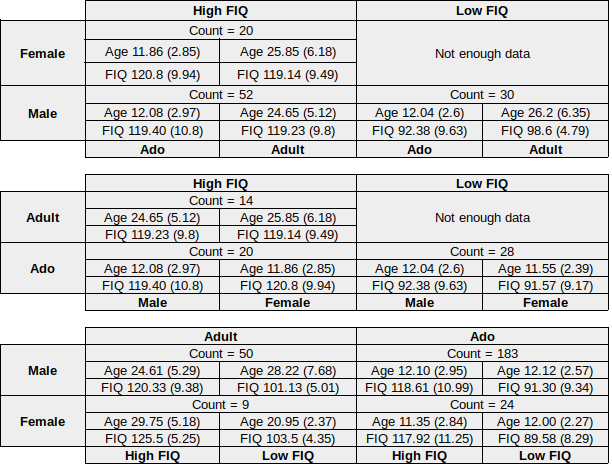


**Supplementary table 4:** characteristics of the three emerged clusters

|  | **Sex (male/female)** | **Age** | **FIQ** |
| --- | --- | --- | --- |
| **Group 1** | 65/10 | 14.99(6.73) | 103.94(16.4) |
| **Group 2** | 87/10 | 15.18(7.77) | 106.7(15.97) |
| **Group 3** | 81/13 | 15.32(6.42) | 107.67(15.6) |


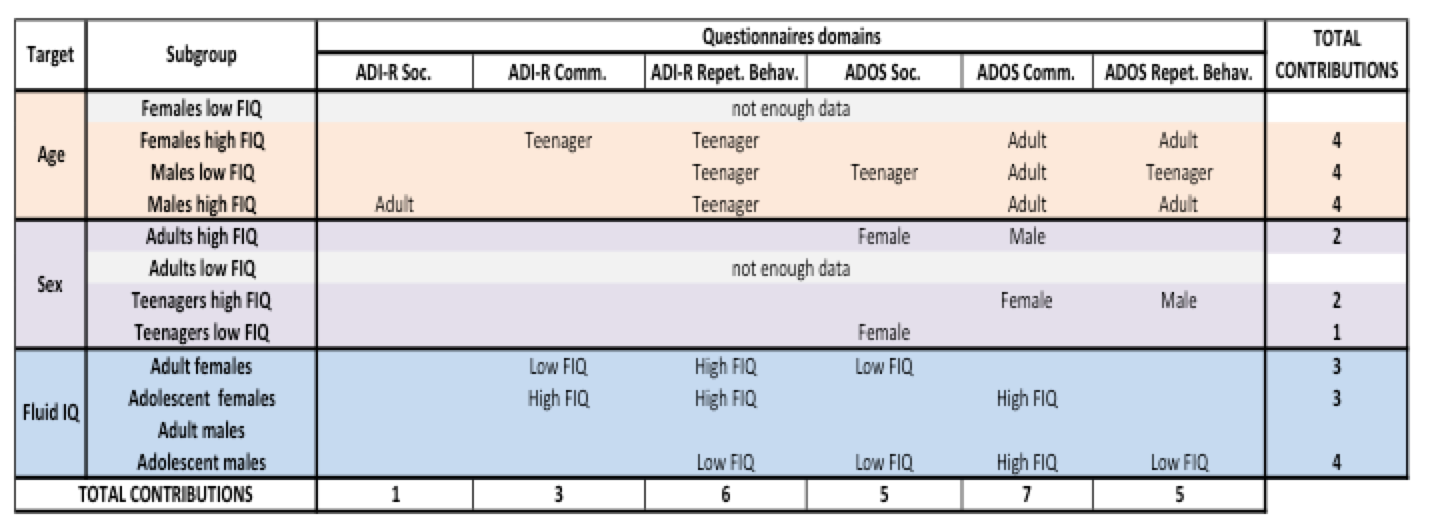
**Supplementary table 5:** Domain contributions to predict age, sex and FIQ per subgroup

Descriptive summary of the results from the logistic regression analyses (Fig. 3-5). Each colored row details the prediction per target of interest for each stratified patient group. Only the domains with important weight per estimation and exhibiting mostly one-sided confidence interval were included in the table. More concretely, we included a domain if two conditions were met: 1) its weight was superior to 80% of the weight average within a subgroup and 2) at least 75% of its confidence intervals must be on the same side. The number of ADI-R and ADOS domains evaluated to carry information about the patients' age (*orange row*), sex (*purple row*), and level of FIQ (*blue row*) are depicted per subgroup (*far right column*) and per instrument domain (*bottom row*). Our results indicate that the communication domain of the ADOS was, on average, the most often used domain to distinguish sex, age and FIQ (7 contributions). The second most informative domain was the repetitive behavior domain of the ADI-R (6 contributions) followed by the social and repetitive behavior domains of the ADOS (5 contributions each). The ADOS domains were the most salient features to estimate patients' sex.

**5. SUPPLEMENTARY FIGURES**

**Supplementary figure 1**


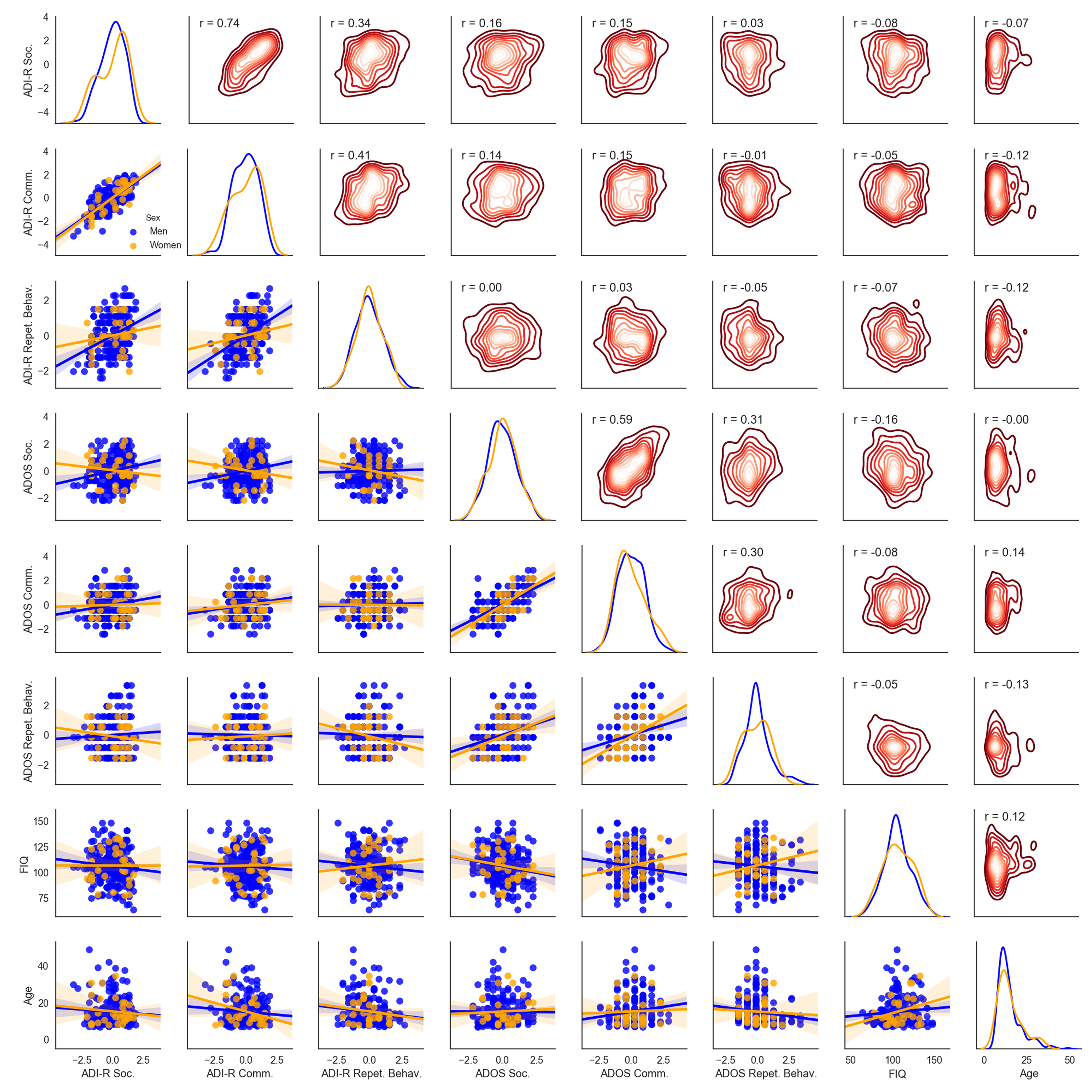
**Distribution of instrument responses by ADI-R and ADOS domains**

Domain scores of the ADI-R and ADOS (social, communication and repetitive behavior) as well as the age, sex and fluid IQ (FIQ) of each patient are showed with their mutual linear relationships. *Diagonal:* The curves represent the distribution of participant scores for each domain. The blue (orange) curve describes the distribution of the male (female) patients. *Lower-left triangle:* The scatter plots display the linear dependencies between each pair of variables. *Upper-right triangle:* Plots the density estimates between each variable (with the correlation coefficient r). The social and communication domains of the ADI-R are quite positively correlated; so are the social and communication domains of the ADOS in both sexes. Across comparisons, a small negative relation was often found between the patients' age and the domain scores of the two instruments. A similar pattern was found between the patients’ FIQ and the instrument scores. When looking at the whole group of patients, the distributions of the ADI-R and ADOS scores as well as the distributions of the age and FIQ were similar in males and females. Overall, the scores of the ADI-R domains seem to be less linearly related to the scores of the ADOS domains, suggesting that both instruments describe complementary aspects of autism symptomatology.

**Supplementary figure 2**
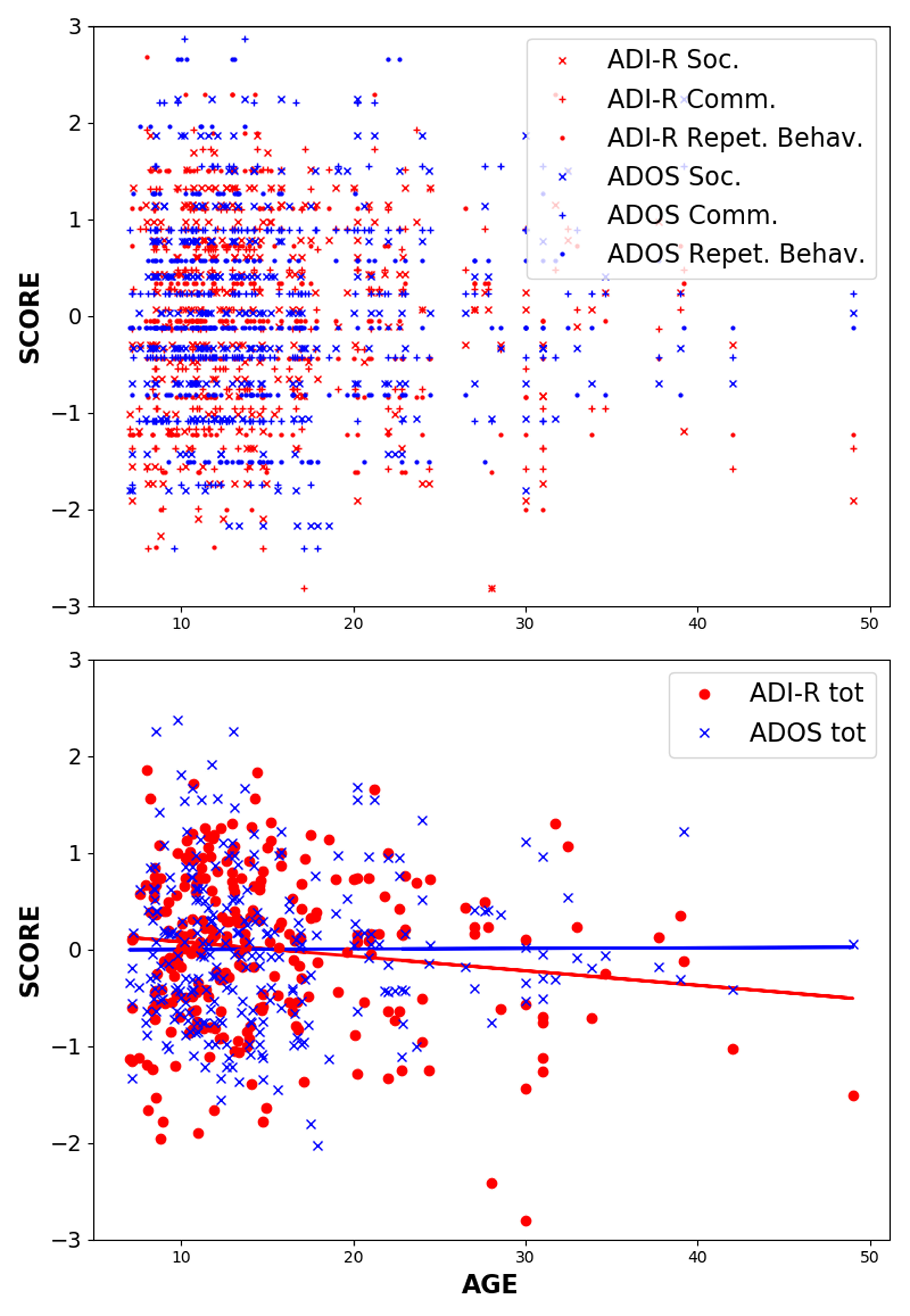


**Score distribution of the ADI-R and ADOS domains by patients' age.**

Domain scores of the ADI-R and ADOS (social, communication and repetitive behavior) across patients' age after standardization.

The plot on the top depicts the scores of the social, communication and repetitive behavior domains of the ADI-R (red crosses, red positive signs, and red points) and the ADOS (blue crosses, blue positive signs, and blue points) across patients' age. The plot at the bottom depicts the mean scores of the three domains of the ADI-R (red circles) and the ADOS (blue crosses) across patients' age. The linear regression lines are plotted for the ADI-R (red line) and the ADOS (blue line) mean scores. In sum, as the patients' age increases, the distribution of the ADOS scores gets sparser but remains homogeneous while the distribution of the ADI-R scores decreases slightly.

**Supplementary figure 3**


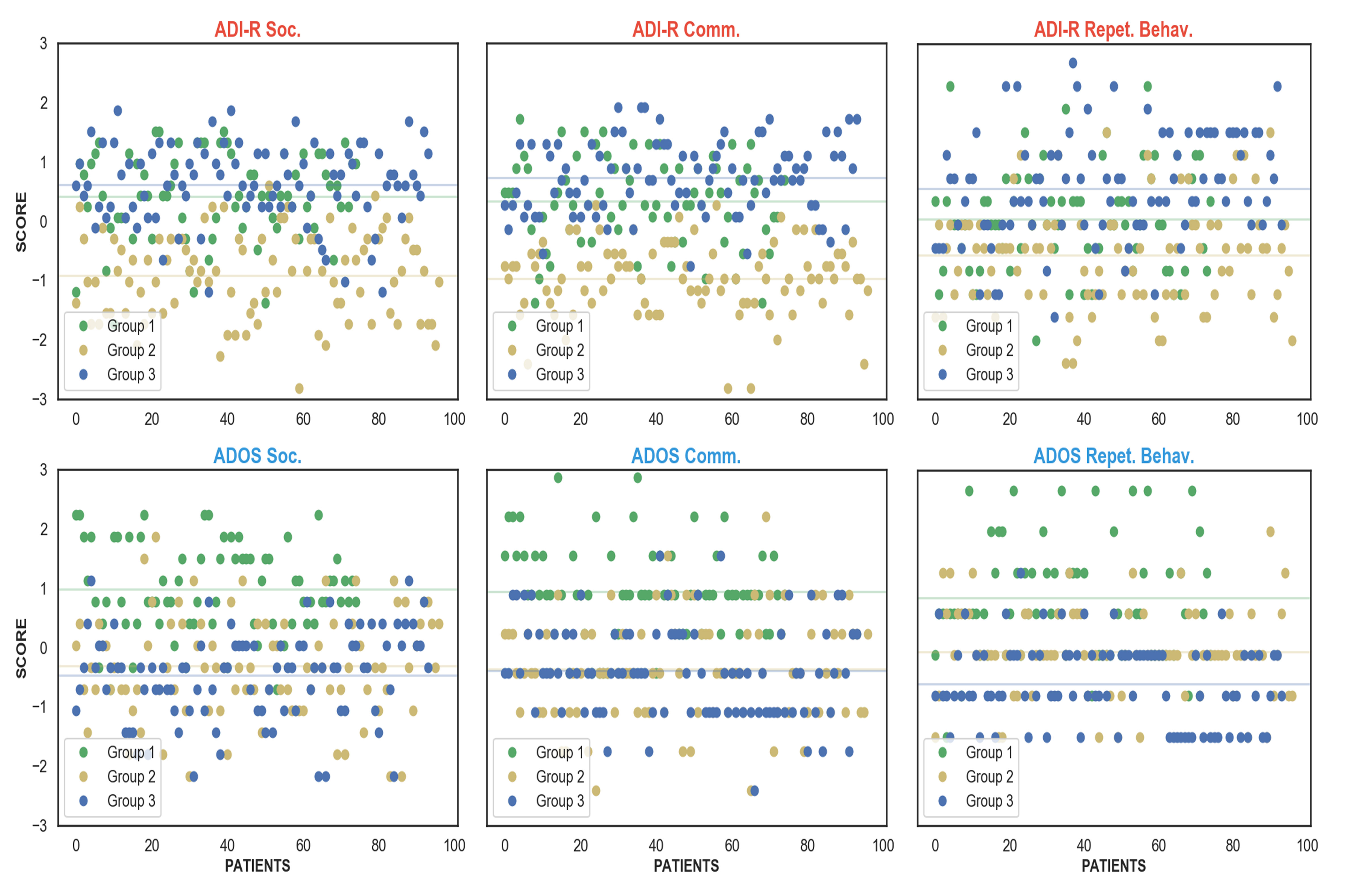


**Exploration of the three extracted patient clusters**

Three patient groups were extracted from the data using automatic clustering (Fig. 1). Each circle represents how a patient scored (*y-axis*) on the specific domain (*six panels*) for a specific patient (*x-axis*). The circle’s color displays what cluster the patient belongs to. Each line shows the mean domain score for a specific cluster of patients (green for group 1, yellow for group 2, and blue for group 3). The social and communication domains of the ADI-R clearly distinguish the second group of patients (yellow). In contrast, the social and communication domains of the ADOS clearly distinguish the first group of patients (green). In the two instruments, the repetitive behavior domain was the least discriminative domain between the three patient groups.

**Supplementary figure 4**


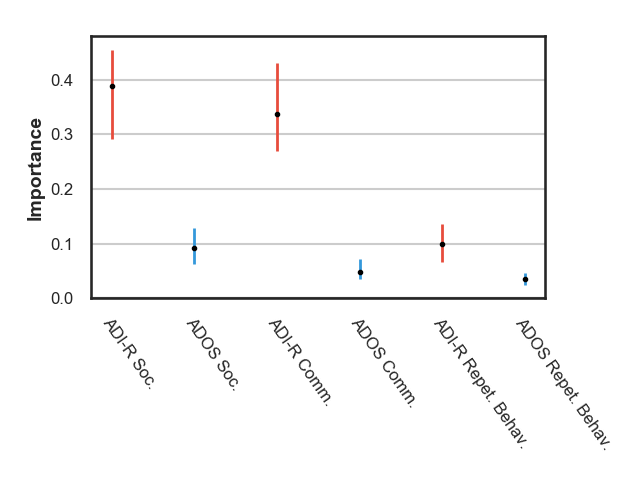


**ADI-R and ADOS domain importance in predicting autism symptom severity**

The random-forest algorithm was used to evaluate the importance of features for discriminating between mild and severe autism. The three domains of the ADI-R and those of the ADOS were used as input to form the predictions. The model achieved an out-of-sample accuracy as high as 92.78% (+/- 0.04 across cross validation folds). The black dots show the feature importance emerging from the set of fitted decision trees. The red bars indicate the bootstrapped 90% uncertainty interval for the importance of the ADI-R domains, while the blue bars represent the bootstrapped 90% uncertainty interval for the importance of the ADOS domains. The plot suggests that the social and communication domains of the ADI-R were most often selected as being the most effective to distinguish patients of different autism severity.

**Supplementary figure 5**


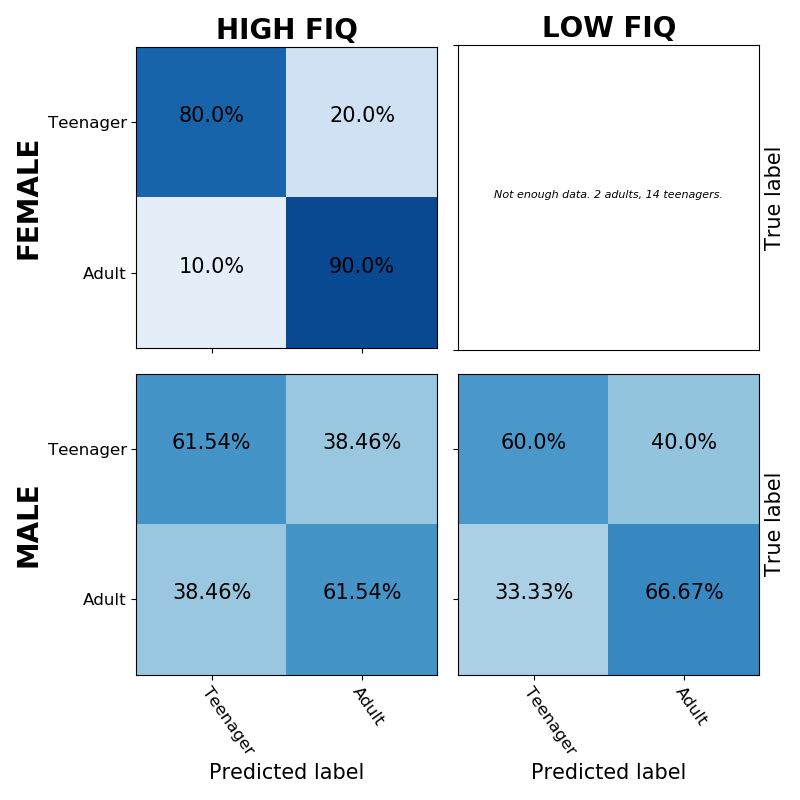


**Confusion matrix of the age prediction segregated by sex and FIQ: classification percentages**

Depicts the confusion matrix for classifying age in females and males with high or low FIQ. For a given confusion matrix, the y-axis shows each of the two targets (*adult or teenager*) while the x-axis shows each of the two predicted labels. The *upper left* and *lower right squares* display the percentage of correct classification while the *upper right* and *lower left squares* display the percentage of misclassification.

For example, in the subgroup of females with high FIQ, 90% of adult participants and 80% of teenager participants were correctly classified by the model (i.e., the logistic regression). In each case, the percentage of correctly classified participants was higher than the percentage of misclassified participants suggesting a good performance of the model. However, the model performed better in female than in male participants.

**Supplementary figure 6**
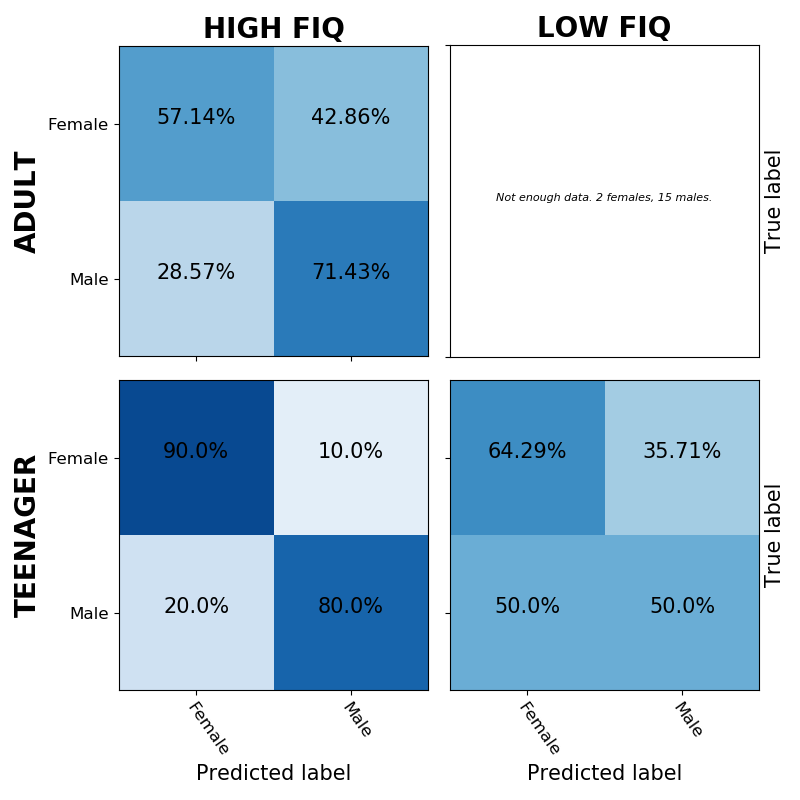


**Confusion matrix of the sex prediction segregated by IQ and age: classification percentages**

Depicts the confusion matrix for classifying sex in adults and teenagers with high or low FIQ. For a given confusion matrix, the *y-axis* shows each of the two targets (*male or female*) while the *x-axis* shows each of the two predicted labels. The *upper left* and *lower right squares* display the percentage of correct classification while the *upper right* and *lower left squares* display the percentage of misclassification.

For example, in the subgroup of adults with high FIQ, 57.14% of females and 71.43% of males were correctly classified by the model (i.e., the logistic regression). In each case, the percentage of correctly classified participants was higher than the percentage of misclassified participants except for predicting males in teenagers with low FIQ suggesting that the score of the ADI-R and ADOS domains are not really relevant to assess patients' sex in this subgroup.

**Supplementary figure 7**
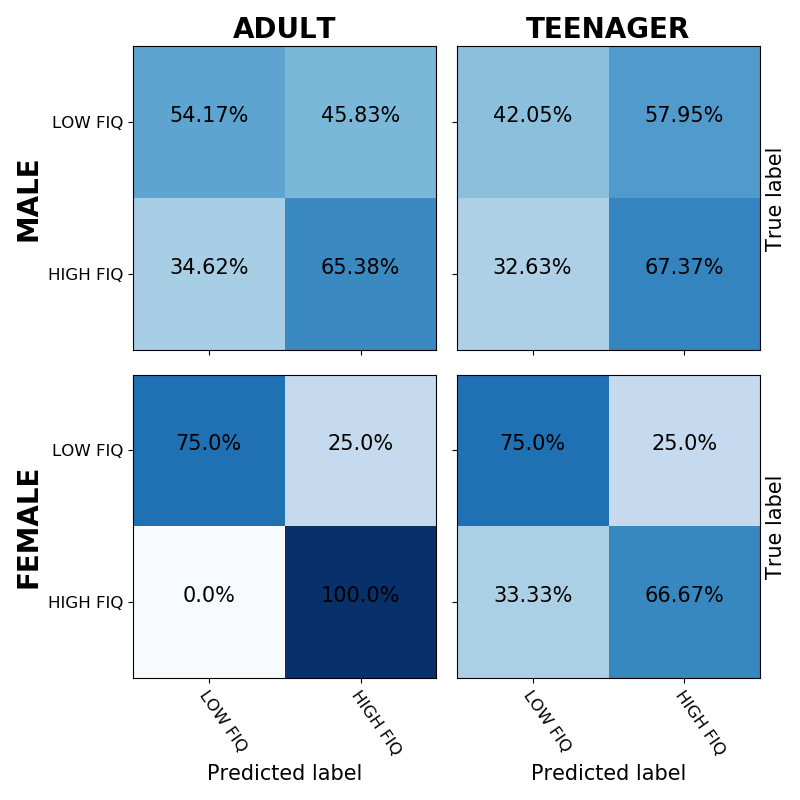


qqqqqq

**Confusion matrix for the prediction of the FIQ segregated by sex and age: classification percentages**

Depicts the confusion matrix for classifying FIQ in male and female adults or teenagers. For a given confusion matrix, the *y-axis* shows each of the two targets (*High* or *low FIQ*) while the *x-axis* shows each of the two predicted labels. The *upper left* and *lower right squares* display the percentage of correct classification while the *upper right* and *lower left squares* display the percentage of misclassification.

For example, in the subgroup of adult males, 54.17% of participants with low IQ and 65.38% of participants with high IQ were correctly classified by the model (i.e., the logistic regression). In each case, the percentage of correctly classified participants was higher than the percentage of misclassified participants except for predicting high IQ in male teenagers suggesting that the score of the ADI-R and ADOS domains are not very informative to classify FIQ in male teenagers.

**6. SUPPLEMENTARY BIBLIOGRAPHY**

Breiman, L. (2001). Random forests. *Machine learning, 45*(1), 5-32.

Chen, C. P., Keown, C. L., Jahedi, A., Nair, A., Pflieger, M. E., Bailey, B. A., & Müller, R.-A. (2015). Diagnostic classification of intrinsic functional connectivity highlights somatosensory, default mode, and visual regions in autism. *NeuroImage: Clinical, 8*, 238-245.

Efron, B., & Tibshirani, R. J. (1994). *An introduction to the bootstrap*: CRC press.

Feczko, E., Balba, N., Miranda-Dominguez, O., Cordova, M., Karalunas, S., Irwin, L., . . . Painter, J. G. (2018). subtyping cognitive profiles in Autism Spectrum Disorder using a Functional Random Forest algorithm. *Neuroimage, 172*, 674-688.

James, G., Witten, D., Hastie, T., & Tibshirani, R. (2013). *An introduction to statistical learning* (Vol. 112): Springer.

Pedregosa, F., Varoquaux, G., Gramfort, A., Michel, V., Thirion, B., Grisel, O., . . . Dubourg, V. (2011). Scikit-learn: Machine learning in Python. *Journal of Machine Learning Research, 12*(Oct), 2825-2830.
